# Application of DFT Simulation to the Investigation of Hydrogen Embrittlement Mechanism and Design of High Strength Low Alloy Steel

**DOI:** 10.3390/ma16010152

**Published:** 2022-12-23

**Authors:** Xiuru Fan, Zhishan Mi, Li Yang, Hang Su

**Affiliations:** Material Digital R&D Center, China Iron & Steel Research Institute Group, Beijing 100081, China

**Keywords:** DFT simulation, hydrogen embrittlement, hydrogen dissolution energy, HSLA steels, fatigue crack propagation

## Abstract

In this work, first-principles methods were performed to simulate interactions between hydrogen and common alloying elements of high strength low alloy (HSLA) steel. The world has been convinced that hydrogen could be one of the future clean energy sources. HSLA steel with a balance of strength, toughness, and hydrogen embrittlement susceptibility is expected for application in large-scale hydrogen storage and transportation. To evaluate the property deterioration under a hydrogen atmosphere, hydrogen embrittlement (HE) of HSLA steel attracts attention. However, due to the small size of hydrogen atoms, the mechanism of HE is challenging to observe directly by current experimental methods. To understand the HE mechanism at an atomic level, DFT methods were applied to simulate the effects of alloying elements doping in bcc-Fe bulk structure and grain boundary structure. Furthermore, the potential application of DFT to provide theoretical advice for HSLA steel design is discussed.

## 1. Introduction

The conflict between resource protection and increasing energy consumption worldwide requires further development and application of renewable energy. Meanwhile, most renewable energies such as solar and wind power are restricted by environmental factors [1]. As a clean, renewable energy and energy storage media, hydrogen attracts attention recently for its high chemical energy density (142 MJ/kg), which is three times larger than petrol. However, as the lightest molecule, the storage and the transport of hydrogen limits the large-scale application. Improving the storage pressure of hydrogen gas can evocatively increase the storage density and efficiency of hydrogen gas.

Currently, austenitic stainless steels are widely accepted as the candidate materials for hydrogen energy-related equipment. However, the low strength grade of austenitic stainless steels could not fulfill the increasing hydrogen storage pressure. High strength low alloy (HSLA) steels have been widely applied for transporting oil and gas [2]. With a superior combination of strength, toughness, and weldability, HSLA steel is also considered as the candidate material for gaseous hydrogen storage and transportation. However, the ductility of conventional metal materials, especially in high strength steels, degrades under hydrogen environment [3]. This phenomenon is commonly known as hydrogen embrittlement (HE) [4]. The hydrogen embrittlement phenomenon seriously deteriorates the mechanical properties of most structural steels, which might directly threaten the safety of large-scale hydrogen applications [5]. 

Multiple factors were suspected to be responsible for the HE of materials, such as the strength of materials, residual stress in materials, concentration of hydrogen traps, heat treatment et al. [6,7,8]. Almost all the influential factors are affected by the chemical composition and microstructure of the material. Experimental methods have been tried to investigate the hydrogen embrittlement mechanism. However, due to the small size of hydrogen atoms, the mechanism of HE is challenging to observe directly by current experimental methods [9,10,11,12]. Therefore, a comprehensive investigation of the interaction between hydrogen and low alloy steels via computational simulation at an atomic scale is expected to improve the understanding of the HE of materials. 

First-principles computational method based on density functional theory (DFT) is an effective method for theoretically studying the physical and chemical properties of materials at the atomic scale, that has been applied broadly to describe the electronic and atomic structures of materials at the atomic level in recent decades [13,14,15,16,17]. DFT calculation could simulate the interaction between hydrogen and alloy atoms and further be applied to investigate the mechanism of HE at the atomic level [18,19,20]. However, current first-principles research of HE mainly concentrated on describing and explaining the interactions of hydrogen with particular alloy elements in specific structures [21,22]. Lacking comprehensive interatomic investigation of common alloy elements underestimates the value of DFT simulation in HE investigation and design of HSLA steels.

In this research, two model plates of steel were characterized under high-pressure hydrogen gas to estimate the hydrogen embrittlement susceptibility. To understand the hydrogen embrittlement mechanism of the two model materials at an atomic level, the interactions of hydrogen and alloying elements in bcc-Fe were systemically studied utilizing first-principles simulation. The hydrogen dissolution energies and hydrogen diffusion coefficients in doped bulk structure and grain boundary were calculated. Additionally, the charge transfer and density of states (DOS) analysis were explored in detail. Furthermore, theoretical advice for designing HSLA steel with low hydrogen embrittlement susceptibility is discussed based on the DFT calculation.

## 2. Materials and Methods

### 2.1. Materials and Experiments

Two plates of model steel with a tensile strength higher than 790 MPa were designed and produced. Commercially pure metals melted utilizing a vacuum induction furnace. 50 kg cast ingots were hot processed to 16 mm plates via hot forging and hot rolling, followed by proper thermal treatment. Specimens were cut from the plate materials, wet ground, polished, and etched with HNO_3_- ethanol solution. A FEI Quanta 650 FEG field emission scanning electron microscopy (FE-SEM) was applied for microstructural observation. 

Hydrogen embrittlement susceptibility of designed materials was evaluated utilizing low strain rate tensile tests and fatigue crack propagation tests. Both tensile properties and fatigue crack propagation properties were tested under high-pressure hydrogen gas in comparison with that under nitrogen gas. Uniaxial tensile tests at 2 × 10^−5^/s were performed, and reduction in area and elongation of a smooth tensile specimen under specific hydrogen/nitrogen pressure were measured, respectively. To generate plots of fatigue crack growth rate as a function of stress intensity factor range, pre-cracked specimens were tested using fracture mechanics methods. 

### 2.2. DFT Calculations

The hydrogen dissolution energies and hydrogen diffusion coefficients in bcc-Fe bulk structure and grain boundary were studied by first-principles calculations based on DFT in the Vienna Ab-initio Simulation Package (VASP). The core electrons and the electron exchange were described utilizing the projector augmented wave (PAW) method, and the correlation was modeled with the Perdew–Burke–Ernzerhof (PBE) method. Plane-wave expansions are considered with a cutoff energy of 500 eV (c.f. Figure S1). All the structures were fully relaxed with the residual force on each atom less than 0.02 eV/Å, and the total energy was converged to 10^−6^ eV/atom for the calculations. All calculations were carried out as spin polarized. The Brillouin zone (BZ) was sampled employing Monkhorst–Pack grids with a 5 × 5 × 5 k-point mesh for the bcc-Fe bulk structure (c.f. Figure S1), and a 5 × 5 × 1 k-point mesh for the bcc-Fe Σ5 GB. The optimized lattice constants are a = b = c = 2.834 Å for the bcc-Fe unit cell (c.f. Table S1), which is consistent with the experimental values a = b = c = 2.86 Å [22].

According to the typical transition state theory, based on the Arrhenius equation, the diffusion coefficient of a hydrogen atom can be calculated as [23,24,25,26]:

(1) $$D(T)=l^{2}\upsilon \;\exp\left(-\frac{E_{diff}}{kT}\right)$$

where l is the hop distance; υ is the attempt frequency; $E_{diff\;}\;$ is the activation energy for the diffusion of a hydrogen atom; k is the Boltzmann constant, and *T* is the temperature. Hop distance (l) and attempt frequency (υ) could be calculated by employing the DFT simulation.

## 3. Results and Discussion

**Figure 1:**
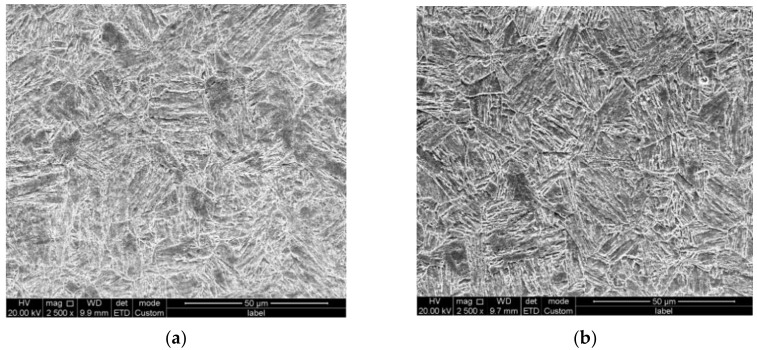
(**a**) Microstructure of model steel 1#, (**b**) microstructure of model steel 2#.

Chemical compositions of the two model materials (1# and 2#) are displayed in Table 1. The microstructures of both materials were characterized by an optical microscope and scanning electron microscope, typical bainite structures were observed in both materials, as displayed in Figure 1. Tensile and impact properties were first characterized to confirm the mechanical properties of the two types of steel after thermal processing. Both tensile and impact test results confirmed the yield strength of the two plates of steel above 790 MPa with sufficient impact absorbing energy (c.f. Table 2). From the mechanical property and microstructure characterization investigation, the two model types of steel were considered qualified.

**Figure 2:**
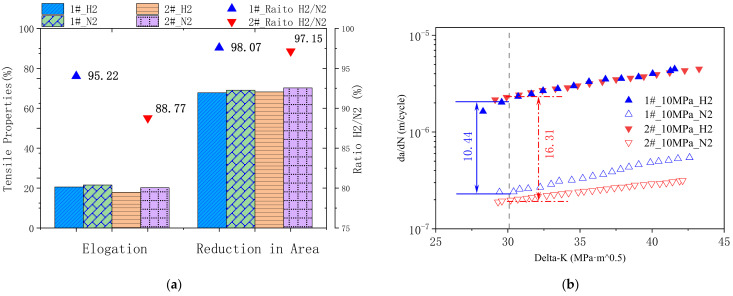
(**a**) low strain rate tensile tests results and (**b**) fatigue crack propagation test results of the two model steels.

The mechanical properties of both model materials under the H_2_/N_2_ pressure at 10 MPa were tested, respectively. Key properties of the low strain rate tensile tests are displayed in Figure 2a. It could be found that the tensile elongation under 10 MPa H_2_ atmosphere for steel 2# decreased by 11.23% compared with that under 10 MPa N_2_ atmosphere. While the tensile elongation determined under H_2_ atmosphere reached over 95% for steel 1#, compared with that under N_2_ atmosphere. Another important tensile property to evaluate hydrogen embrittlement susceptibility is the ratio of reduction in area. In both model materials, the reduction in area under H_2_ gas decreased to 98.07% and 97.15% in comparison to the ones tested in N_2_ gas, respectively. In view of low strain rate tensile tests, steel 1# behaves with less hydrogen embrittlement susceptibility.

It has been found that cracks due to HE might grow rapidly [27]. Thus, the crack propagation properties of model steels were investigated to further compare the HE susceptibility of the two materials. Figure 2b plots the crack growth rate (crack extension per load cycle, da/dN) as a function of the stress intensity factor range(ΔK). The crack growth rate increased with higher stress intensity, and both types of steel behaved at higher crack growth rates in the hydrogen atmosphere. At ΔK = 30, the crack growth rate of steel 1# in hydrogen gas is 10.44 times higher than that in nitrogen gas, while the crack growth rate ratio of steel 2# is 16.31 (Figure 2b). Both fatigue crack propagation tests and low strain tensile tests indicate that steel 1# behaves a less susceptibility of hydrogen embrittlement in high-pressure hydrogen gas.

**Figure 3:**
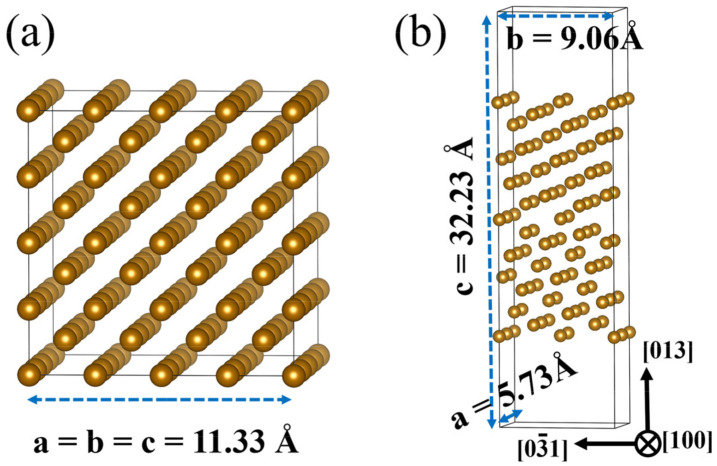
Modelled structure of (**a**) bcc-Fe bulk, (**b**) bcc-Fe Σ5 GB.

To understand the different HE behavior between the model materials from the atomic level, first principles method calculations were performed. We first studied the effects of alloying elements (Cr, Mn, Ni, and Mo atoms), which differ the two materials in chemical composition, on H dissolution energies of bcc-Fe bulk structure and bcc-Fe grain boundary (GB). For the periodic boundary condition, 4 × 4 × 4 bcc-Fe supercell was used to keep the distance between two repeated defects far enough, and thus the interaction between the defects could be ignored. The 4 × 4 × 4 bcc-Fe supercell includes 128 Fe atoms with the optimized lattice constants a = b = c = 11.33 Å, as shown in Figure 3a. The atomic structure diagram of bcc-Fe Σ5 GB was modeled to represent symmetric tilt grain boundaries based on an example of a real boundary with a vacuum layer of 15 Å is shown in Figure 3b [28,29]. In the calculation of doped atoms at the bcc-Fe Σ5 GB, the upper and lower four layers of Fe atoms were fixed. After structural optimization, the lattice constants became a = 5.73 Å, and b = 9.06 Å. 

The H dissolution energies of pure bcc-Fe bulk structure and pure bcc-Fe Σ5 GB were calculated to investigate the effects of alloying elements. The H dissolution energy *E_s_* is defined as:

(2) $$E_{s}=E_{\left(structure\;with\;H\right)}-E_{\left(structure\right)}-\frac{E_{\left(H_{2}\right)}}{2}$$

where *E*_(*structure with H*)_ is the total energy of the pure or doped bcc-Fe bulk structure or GB with an interstitial H atom, and *E*_(*structure*)_ is the total energy of the pure or doped bcc-Fe bulk structure or GB without H atoms. $\frac{E_{\left(H_{2}\right)}}{2}$ refers to the energy of an H atom in a single H_2_ molecule. Hence, a lower H dissolution energy indicates that the structure is more favorable to combine with H atoms.

The H dissolution energies of different locations were first calculated, and the most stable one was chosen for further calculation and discussion. In the bcc-Fe bulk structure, an H atom can occupy an octahedral interstitial site (o-site), or a tetrahedral interstitial site (t-site). The calculated *E_s_* in the o-site is 0.35 eV and *E_s_* in the t-site is 0.22 eV, which is consistent with the results calculated by W. Counts et al. [22]. Thus, we uniformly optimized the structure of the H atom in the t-site for subsequent calculations. For the pure bcc-Fe Σ5 GB model, the calculated *E_s_* is −0.26 eV. A negative value for the H dissolution energy represents an energy release when the H atom diffused into the GB interstitial site. That indicates the H atoms are easily dissolved at grain boundaries, where the HE is more likely to occur [30,31].

**Figure 4:**
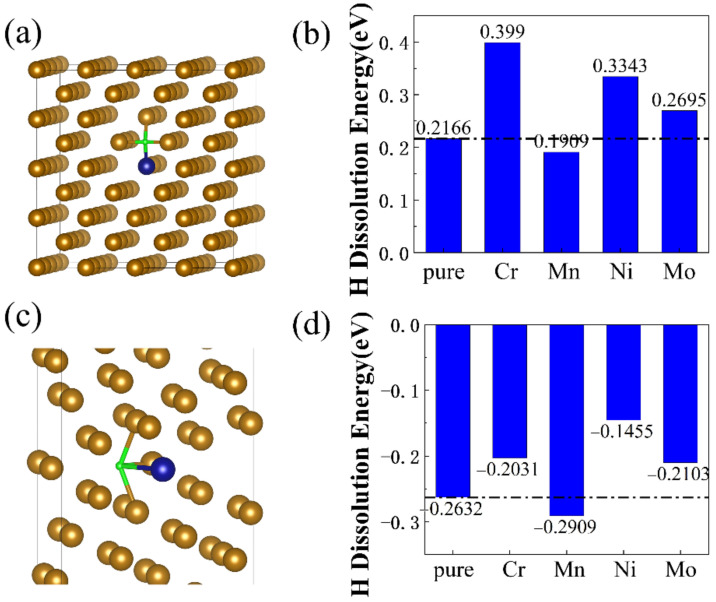
(**a**) doped bcc-Fe bulk structure with H, (**b**) H dissolution energies of doped bcc-Fe bulk structure; (**c**) doped bcc-Fe Σ5 GB with H; (**d**) H dissolution energies of doped bcc-Fe Σ5 GB.

The H dissolution energies of bulk and Σ5 GB structures of bcc Fe with different alloying elements (Cr, Mn, Ni, and Mo atoms) were calculated by Equation (2). One Fe atom in the bcc-Fe bulk/Σ5 GB structure was substituted by Cr, Mn, Ni or Mo atom, respectively, corresponding to the doping concentration of 0.73, 0.77, 0.83 or 1.35% (mass fraction) in bulk structure, and 1.04, 1.10, 1.19 or 1.93% (mass fraction) in Σ5 GB structure. In Figure 4a,c, the blue atoms represent the alloying elements and the green ones represent the interstitial H atoms. Figure 4b,d display the H dissolution energies of bcc-Fe bulk structures and bcc-Fe Σ5 GB structures with alloying elements (Cr, Mn, Ni, and Mo atoms), respectively. The H dissolution energy values range from 0.19 eV to 0.40 eV for bcc-Fe bulk structure with alloying elements, and range from −0.29 eV to −0.15 eV for bcc-Fe Σ5 GB structure with alloying elements.

The positive H dissolution energies, in bcc-Fe bulk structure with alloying elements, indicate that H atoms are either unstable or energetically unfavorable to occupy the lattice interstitial sites. The negative H dissolution energies, in bcc-Fe Σ5 GB structure with alloying elements, confirm that H atoms could stably occupy the interstitial sites at grain boundaries. In comparison to pure Fe structures, the H dissolution energies decreased while doping a Mn atom in bulk and GB structures. Additionally, the H dissolution energies increased in Cr-doped, Ni-doped, and Mo-doped structures. This indicates that Mn addition might lead to easier dissolution of H atoms in both bulk and GB structures.

To further understand the role of alloying elements in hydrogen damage, we studied the impact of alloying elements on hydrogen diffusion. Diffusion paths between different sites considering alloying elements were determined and studied employing the nudged-elastic band (NEB) methods [32,33]. This method is advantageous if the exact position of the transition point is unknown and/or if detailed information about the energy profile along the transition path is needed. Several images between two fully relaxed endpoints were calculated to simulate the diffusion pathway, and each image was relaxed until the perpendicular forces with respect to the minimum energy path were less than a given tolerance, which was set to 0.05 eV/Å in this case. 

**Figure 5:**
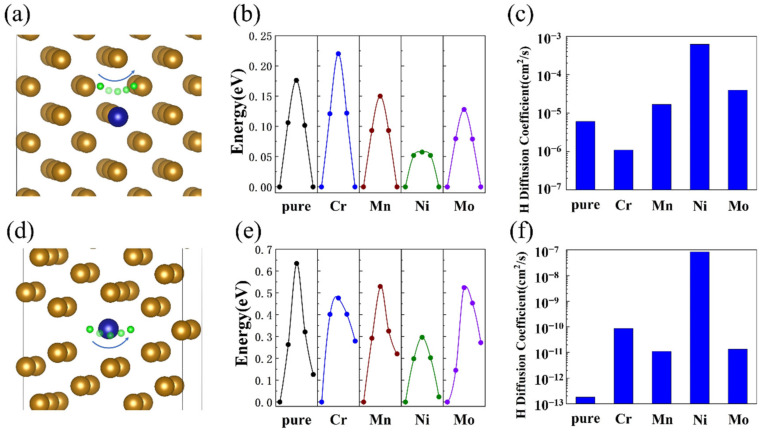
(**a**) hydrogen diffusion in doped bcc-Fe bulk structure, (**b**) H diffusion barriers of doped bcc-Fe bulk structure, (**c**) H diffusion coefficients of doped bcc-Fe bulk structure, (**d**) hydrogen diffusion in doped bcc-Fe Σ5 GB, (**e**) H diffusion barriers of doped bcc-Fe Σ5 GB, (**f**) H diffusion coefficients of doped bcc-Fe Σ5 GB.

The diffusion of H atoms within the bcc-Fe bulk structure was investigated by employing CI-NEB calculations. H atoms at the interstitial site diffused to its equivalent site along the relax diffusion path, as illustrated in Figure 5a. For the hydrogen diffusion in pure bcc-Fe bulk structure, we obtained a diffusion barrier of 0.17 eV, as shown in Figure 5b, which is consistent with ab initio molecular dynamics simulation reported by Sanchez et al. [34] This indicates that the predicted hydrogen diffusion path is reasonable. The diffusion coefficient of a hydrogen atom in a pure bcc-Fe bulk structure can be estimated according to Equation (1):

(3) $$D\left(T\right)=5.73\times 10^{-3}{\mathrm{cm}}^{2}/s\;\exp\left(-2043/T\right)$$

The calculated hydrogen diffusion coefficient at room temperature (*T* = 298 K) is 6.03 × 10^–6^ cm^2^/s, which is consistent with the literature value [35]. 

Meanwhile, the diffusion of H atoms within the bcc-Fe Σ5 GB structure was investigated. Figure 5d displays the schematic diagram of a hydrogen diffusion path in a bcc-Fe Σ5 GB structure with alloying elements. Figure 5e,f show the calculated diffusion barriers and diffusion coefficients of pure and doped bcc-Fe Σ5 GB structure. A diffusion barrier of 0.63 eV for the hydrogen diffusion was obtained within the pure bcc-Fe Σ5 GB. The diffusion coefficient of a hydrogen atom in a pure bcc-Fe Σ5 GB structure can be estimated according to Equation (1):

(4) $$D\left(T\right)=9.73\times 10^{-3}cm^{2}/s\;\exp\left(-7366/T\right)\;$$

Thus, the hydrogen diffusion coefficient of Σ5 GB at room temperature (*T* = 298 K) was calculated to 1.79 × 10^–13^ cm^2^/s. This indicates that H atoms diffuse much slower in grain boundaries than in inner grains. 

In comparison to bulk structures, GB structures show a higher diffusion barrier of H atoms (c.f. Figure 2 and Figure 3). Xiao Zhou et al. presumed that H diffusion at GBs might be influenced by the different lattice patterns of the tilt axis because of the two-dimensional nature of GBs [36,37]. Meanwhile, the H diffusion depends on the alloying element in the bulk structure is relevantly equivalent. The structural differences between GB and bulk structures lead to the lower dissolution energy and higher diffusion barrier and behave as the slower H diffusion at grain boundaries. The slower diffusion and lower dissolution energy of hydrogen atoms at the grain boundaries might lead to the segregation of hydrogen atoms at the grain boundaries, which could lead to hydrogen embrittlement. 

The hydrogen diffusion barriers and diffusion coefficients in bulk and GB structures with alloying elements were calculated. As shown in Figure 5b,c, the diffusion of H atoms inside the Cr-doped bcc-Fe bulk structure needs to cross a higher energy barrier than in pure Fe of 0.22 eV. On the other hand, the hydrogen diffusion barriers decreased for Mn-doped, Ni-doped, and Mo-doped bulk structures. The diffusion coefficient of H varied from 1.09 × 10^–6^ cm^2^/s (for Cr-doped) to 6.16 × 10^–4^ cm^2^/s (for Ni-doped) with doping alloy elements. The diffusion coefficients of the GB structure with alloying elements at room temperature kept slower than in bulk structures. However, the hydrogen diffusion coefficients in bcc-Fe Σ5 GB can be increased by orders of magnitude while doping alloying elements, especially by nickel (9.46 × 10^–8^ cm^2^/s). Thus, the addition of alloying elements might contribute to the reduction of hydrogen accumulation, and benefit the properties of materials under hydrogen atmosphere.

To further understand the interaction of hydrogen and alloying elements and the influence of such interaction on the hydrogen embrittlement susceptibility, charge transfer, and density of states (DOS) analysis were explored. The alloying elements Cr and Mn doping in bcc-Fe Σ5 GB are exampled for illustration in detail.

The valence electron distribution of H in doped structures was analyzed utilizing the Bader Charge Analysis program code. The valence electron numbers of H in Cr and Mn doped structure are 1.29 e and 1.35 e, respectively. Which indicates that in Mn doped structure H gains more electrons and has strong interaction with the structure.

The differential charge density of H in Cr and Mn doped structure is defined as:

(5) $$\rho =\rho_{\left(structure\;with\;H\right)}-\rho_{\left(structure\right)}-\rho_{\left(H\right)}$$

where *ρ*_(*structure with H*)_ is the total charge density of the doped structure with an H atom; *ρ*_(*structure without H*)_ and *ρ*_(*H*)_ are the charge density of the doped structure and H atom, respectively. 

**Figure 6:**
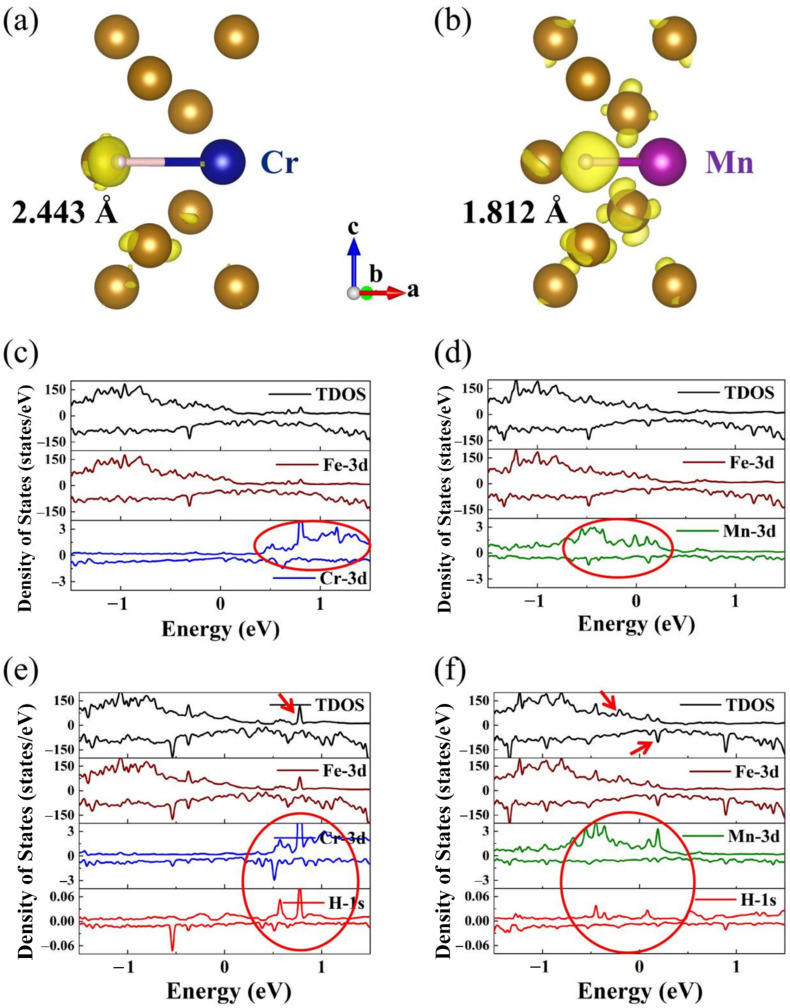
(**a**) Differential charge density of H in Cr doped structure; (**b**) Differential charge density of H in Mn doped structure; The charge density iso-surface is set to be 0.005 e/Å^3^; (**c**) The density of states with spin-up and spin-down for Cr doping in bcc-Fe Σ5 GB without H atom; (**d**) The density of states with spin-up and spin-down for Mn doping in bcc-Fe Σ5 GB without H atom; (**e**) The density of states for Cr doped structure with H atom; (**f**) The density of states for Mn doped structure with H atom.

Figure 6a,b display the illustration of charge density of H in the Cr and Mn doped structure. The iso-surface of charge density is set to 0.005 e/Å^3^. The yellow areas represent valence electrons increment of H atom. The H atom around doping atom Mn gains more valence electrons than that around doping atom Cr, which results in a more restrained bonding of H to Cr atoms. Moreover, the Mn-H bond length (1.812 Å) is shorter than the Cr-H bond length (2.443 Å). Similarly, the H atom around doping atom Ni and Mo also gain fewer valence electrons with longer bond length.

The total density of states (TDOS) and partial density of states (PDOS) with spin-up and spin-down for Cr and Mn doping in bcc-Fe Σ5 GB with and without H atom are illustrated in Figure 6c–f. The positive values represent the spin-up electron states, the negative values represent the spin-down electron states, and the energy 0 eV represents the Fermi level. The doped structures without H exhibit metallic properties and have no band gap from Figure 6c,d. The Fe-3d (*d_xy_, d_yz_, d_xz_, d_x_*_2_, and *d_z_*_2_) states are more pronounced below the Fermi level for the Cr- and Mn-doped structure. For doping atom Cr, the Cr-3d states mainly contribute more than 0.5 eV above the Fermi level, while Mn-3d states mainly have a contribution around the Fermi level, from −0.5 eV to 0.2 eV. Thus, the Fe-3d states and Mn-3d states have more overlaps, which indicates an increase in Fe and Mn d orbital hybridization. For doped structure with an H atom, the contributions to the energy level of Fe-3d states, Cr-3d states, and Mn-3d states are almost equal to that without H atoms from Figure 6e,f. The Fe-3d states are more pronounced below the Fermi level, the doping atom Cr-3d states mainly contribute above the Fermi level more than 0.5 eV, and the doping atom Mn-3d states mainly have a contribution around the Fermi level. 

Furthermore, for Cr doped structure with an H atom, the H-1s states mainly contribute above the Fermi level from 0.5 eV to 0.8 eV, and for Mn doped structure with an H atom, the H-1s states mainly contribute around the Fermi level. It is worth noting that the electronic density of states has enhanced due to the introduction of the hydrogen atom. In the ground state, the valence bands below the Fermi level (represented as 0 eV in Figure 6) are filled with electrons, and the conduction bands above are empty. The TDOS of Cr doping structure with H atom presents enhanced peaks at an energy of around 0.8 eV (c.f. Figure 6e, red arrow), which is located at empty bands. While in Mn doping structure with H atom, enhanced peaks appear at the energy from about −0.2 eV to 0.2 eV (c.f. Figure 6f, red arrows) and the DOS peaks below 0 eV would exhibit strong hybridization. The hybridization of H-1s states with Fe-3d states and Mn-3d states in Mn doping structure might enhance the interaction of H and Mn atoms in bcc-Fe Σ5 GB.

Alloying elements Cr, Ni, and Mo doping in bcc-Fe bulk and Σ5 GB structures could increase H dissolution energies while alloying element Mn doping would decrease H dissolution energies. Moreover, doping alloy elements in the bulk structures could lead to the variation of H diffusion coefficients, but less pronounced than that in the Σ5 GB structures. According to the DFT analysis, in materials with a bcc-Fe matrix, the addition of Ni, Cr, and Mo might benefit the properties under hydrogen atmosphere, while Mn addition might deteriorate the properties of α-Fe based steel. That explains that model steel 1# with higher Ni, Cr, and Mo contents and lower Mn content might behave with lower hydrogen embrittlement susceptibility than model steel 2#. Furthermore, since Ni, Cr, Mo, and Mn are the most commonly used alloy elements in HSLA steel, outcomes of this work could provide theoretical advice for the designing HSLA steel with low HE hydrogen embrittlement susceptibility.

## 4. Conclusions

In summary, the hydrogen embrittlement susceptibility of two model materials was characterized under high-pressure hydrogen gas. The interaction of hydrogen and alloying elements (Cr, Mn, Ni, and Mo atoms) in bcc-Fe bulk and at grain boundary were investigated utilizing first-principles calculations. The main outcomes are summarized as follows:

(a) Model steel 1# with higher content of Ni, Cr, and Mo behaves with lower susceptibility of hydrogen embrittlement in high-pressure hydrogen gas;
(b) Alloying elements Cr, Ni, and Mo doping in bulk and Σ5 GB structures could increase H dissolution energies, while Mn doping could decrease H dissolution energies;
(c) In comparison to bulk structures, GB structures show a higher diffusion barrier of H atoms and a lower H diffusion coefficient at grain boundaries;
(d) Alloying elements significantly affect the hydrogen diffusion behavior, especially at Σ5 GB. The hydrogen diffusion coefficient for Ni dopingΣ5 GB structures increases by approximately six orders of magnitude.

Based on the DFT simulation, alloying elements Cr, Ni, and Mo might benefit the properties under hydrogen atmosphere. Additionally, Mn addition might deteriorate the properties of α-Fe based steel. DFT simulation can improve the understanding of the HE mechanism of HLSA steel and provide theoretical advice for alloy design.

## Figures and Tables

**Table 1:** Chemical composition of model steels.

	C (wt%)	Si (wt%)	Mn (wt%)	P (wt%)	S (wt%)	Ni (wt%)	Cr (wt%)	Mo (wt%)	V (wt%)	Fe
1#	0.12	0.2	1.02	<0.01	0.001	1.12	0.42	0.47	0.03	R
2#	0.14	0.19	1.38	<0.01	0.002	0.30	0.22	0.24	0.03	R

**Table 2:** Mechanical properties of model steels.

	*YS/MPa*	*UTS/MPa*	*KV_2_ (−40)/J*
*1#*	792.5	843	219.3
*2#*	804.5	844	189

## Data Availability

Not applicable.

## Supplementary Materials

The following supporting information can be downloaded at: https://www.mdpi.com/article/10.3390/ma16010152/s1, Figure S1: (a) The convergence tests for the plane-wave energy cut-off. (b) The convergence tests for the Monkhorst-Pack k-point grids; Table S1: Lattice constant calculated by different methods in comparison to experimental value.
