# Supplementary material for: Application of DFT Simulation to the Investigation of Hydrogen Embrittlement Mechanism and Design of High Strength Low Alloy Steel
Source: Materials (Basel). 2022 Dec 23;16(1):152. doi: 10.3390/ma16010152 (PMC9821312; doi:10.3390/ma16010152)
Supplement: Supplementary file 1 [file materials-16-00152-s001.zip › materials-2054395-supplementary.pdf]

materials

MDPI

Supplementary Materials

Application of DFT Simulation to the Investigation of Hydrogen Embrittlement Mechanism and Design of High Strength Low Alloy Steel

Xiuru Fan, Zhishan Mi *, Li Yang and Hang Su

Material Digital R&D Center, China Iron & Steel Research Institute Group, Beijing 100081, China; fanxiuru@hotmail.com (X.F.); yangli@cisri.com.cn (L.Y.); suhang@cisri.com.cn (H.S.)
* Correspondence: mizhishan@163.com; Tel.: +86-15711270072

Optimized lattice constants of bcc-Fe unit cell obtained by GGA, LDA, and VDW methods were calculated and displayed in experimental values, as shown in Table S1. The value calculated using the GGA method is closest to the experimental one. Therefore, the GGA method was used in this research.

Table S1. Lattice constant calculated by different methods in comparison to experimental value.

Method		Lattice Constant /Å
**Calculational**	GGA	2.834
	LDA	2.749
	VDW	2.807
Experimental		2.86

Convergence tests were conducted for the plane-wave energy cut-off and Monkhorst-Pack k-point grids, as shown in Figure S1. The total energy of the bcc-Fe bulk structure is almost constant with the Monkhorst-Pack k-point grids from 3 to 7 and plane-wave energy cut-off from 400 eV to 600 eV. Thus, the plane-wave cut-off energy was set to 500 eV and the Monkhorst-Pack k-point grids were set to 5 × 5 × 5.

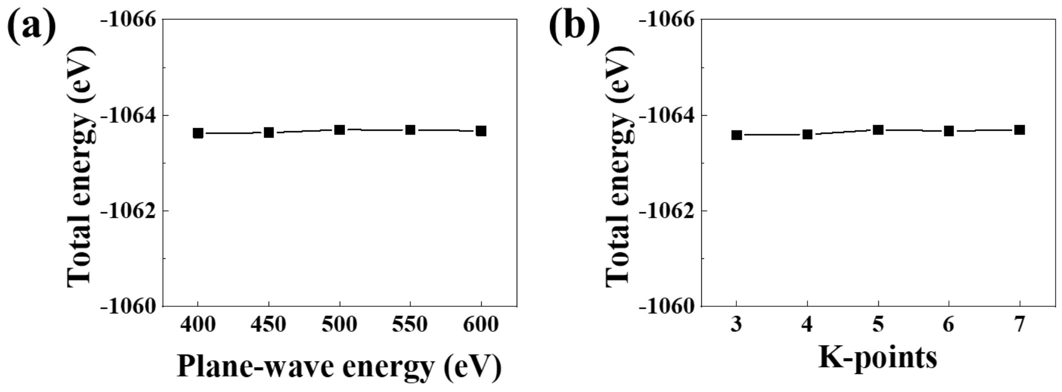

Figure S1. (**a**) The convergence tests for the plane-wave energy cut-off. (**b**)The convergence tests for the Monkhorst-Pack k-point grids.
